# Cerebral venous sinus thrombosis as the initial presentation of essential thrombocythemia - A case report and literature review

**DOI:** 10.1016/j.ensci.2022.100398

**Published:** 2022-03-31

**Authors:** Qudsum Yousaf, Haseeb Amad Khan, Fateen Ata, Adeel Ahmad Khan, Nadia Karim, Zohaib Yousaf

**Affiliations:** a Department of Neurology, Central Park Teaching Hospital, Lahore, Pakistan; b Department of Internal Medicine, Nishtar Medical College and Hospital, Multan, Pakistan; c Department of Internal Medicine, Hamad General Hospital, Hamad Medical Corporation, Doha, Qatar; d Department of Endocrinology, Hamad General Hospital, Hamad Medical Corporation, Doha, Qatar

**Keywords:** Myeloproliferative disorders, Cerebral venous sinus thrombosis, Essential thrombocythemia, Stroke

## Abstract

Myeloproliferative disorders (MPD) are associated with vascular thrombosis. Common sites for thrombosis are large arteries; however, less commonly, cerebral venous sinus thrombosis (CVST) has also been reported. It is rare to have CVST as an initial presentation of MPD. We discuss a male patient in whose presentation due to CVST led to the diagnosis of essential thrombocythemia (ET). Furthermore, we performed a literature review to evaluate the association of CVST with ET.

## Highlights

• Myeloproliferative disorders can rarely manifest as cerebral venous sinus thrombosis with its initial presentation.
• Literature review suggests a female preponderance for the development of CVST in MPD.
• Patients usually have a favorable outcome with anticoagulation and cytoreductive therapy.
• The role of novel anticoagulants is emerging in patients with CVST.
• Until more evidence is available, it should be limited where standard anticoagulation cannot be done safely.

## 1 Introduction

Cerebral venous sinus thrombosis (CVST) is an uncommon type of stroke, which is frequent in females (3:1) [1]. It accounts for 0.5% of all strokes [2]. Myeloproliferative disorders (MPD) can rarely present as CVST as their initial manifestation [3]. Typical sites of thrombosis in MPD are large arteries. Among MPD, polycythemia vera and essential thrombocythemia (ET) have shown an association with thrombosis at atypical sites such as cerebral venous sinuses and splanchnic vein [4]. Common mutations in ET are Janus kinase (JAK2) and calreticulin (CALR.). Thrombotic sequelae are more commonly associated with JAK2 mutation [5].

ET is diagnosed based on the World Health Organization criteria [6]. The criteria consist of a sustained platelet count ≥450 × 10^9^/L, the presence of an acquired pathogenetic mutation, absence of other myeloid malignancy, an absence of a reactive cause for thrombocytosis, and normal iron stores. There is an increased production of platelets in ET, which might be responsible for the prothrombotic state leading to thrombosis, e.g., CVST. Prompt diagnosis and management of CVST are crucial for having favorable outcomes in ET [7]. CVST affects 4 per million adults, predominantly affecting adults in their 30s and 40s, and 7 per million pediatric population [8].

We report a 37-year-old presenting with CVST and was later found to have underlying ET. The case is accompanied by a literature review of the previously reported cases on the topic [3,[9], [10], [11], [12], [13]].

## 2 Case presentation

A 37-year-old previously healthy Pakistani male presented with a persistent, moderately severe, bilateral frontal and occipital headache for four months. The headache was not associated with nausea, vomiting, fever, photophobia, or neck stiffness. The patient did not seek medical advice and used over-the-counter analgesics for pain without much relief. The headache was associated with the heaviness of the head. The patient also developed a painless, progressive blurring of vision two weeks after headache onset. The blurring of vision was initially in the left eye and then involved the right eye—no previous similar episodes were noted. There was no associated fever, rash, joint pain, photosensitivity, photophobia, burning micturition, cough, night sweats, diarrhea, or weight loss. A complete review of systems was unremarkable.

Upon presentation, the patient was afebrile (36.3 °C), normotensive (127/83 mmHg), had a heart rate of 83 beats per minute, a respiratory rate of 17 breaths per minute, and saturated well on room air. He was conscious, alert, oriented with a Glasgow coma scale of 15/15. The neurological examination revealed a normal motor, sensory and cerebellar exam. Cranial nerve examination revealed a visual acuity of 6/24 in the right eye and finger counting with a positive rapid afferent pupillary defect (RAPD) in the left eye. Fundoscopy revealed bilateral grade 3 papilledema. The rest of the clinical examination was unremarkable.

Initial investigations, including a complete blood count, electrolytes, renal function test, liver function test, thyroid function test, and coagulation profile, revealed thrombocytosis (1327 × 10^3^ per microliter) and prolonged bleeding time. (Table 1) A peripheral smear confirmed the presence of thrombocytosis along with platelet anisocytosis.

**Table 1:** Laboratory parameters (aPTT = Activated Partial Thromboplastin Clotting Time, INR = international normalized ratio, fT4 = free Thyroxine).

Variable	Values	Reference ranges
White cells (per mm3)	7.6	4.5–10
Platelet count (10^9^/L)	1327	150–400
Hemoglobin (gm/Liter)	15	14–18
ESR (mm/1st hour)	28	1–13
Total Bilirubin (mg/dL)	0.7	0.3–1
Total protein (g/Liter)	78	60–83
Albumin (g/Liter)	41	34–54
Alkaline Phosphatase (Unit/Liter)	215	38–126
Alanine Aminotransferase (Unit/Liter)	27	0–35
Aspartate Aminotransferase (Unit/Liter)	38	17–59
Glucose (mmol/L)	8.2	3.9–5.5
Urea (mmol/L)	3.8	2.1–8.5
Creatinine (Umol/Liter)	62	61.9–114.9
Sodium	134	135–145
Potassium (mmol/L)	3.4	3.6–5.2
Chloride (mmol/L)	102	96–106
Bicarbonate (mmol/L)	23	23–29
Corrected Calcium (mmol/L)	2.23	2–2.5
Bleeding time (minutes, seconds)	2 min, 15 s	2–7 min
aPTT (seconds)	48	21–35
INR	1.1	<1.1
PT (seconds)	15	10–13
TSH (mIU/L)	2.1	0.4–4
fT4 (ng/dL)	1.5	0.7–1.9

CSF (Lumbar Puncture)		
Appearance	Clear	–
Xanthochromia	Negative	–
Red blood cells (number)	Nil	0
White blood cells (number)	Nil	<3
Protein (mg/dL)	20	15–60
Glucose (mmol/L)	4	2.5–3.5

**Fig. 1:**
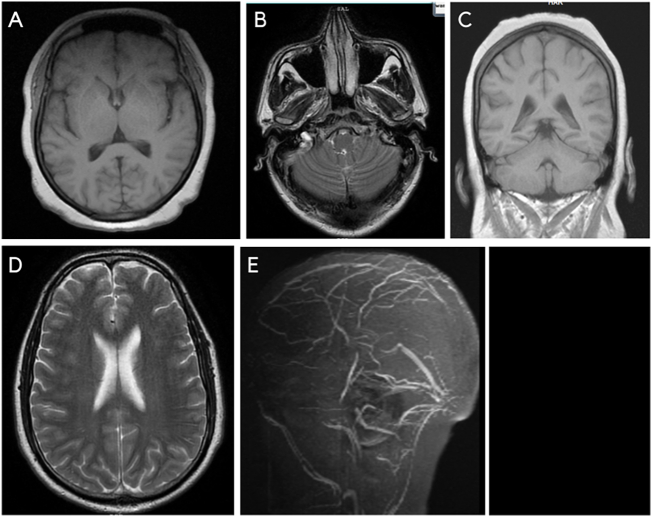
A-E: Magnetic resonance imaging (MRI) and magnetic resonance venography (MRV) images showing extensive cerebral venous sinus thrombosis, including the superior sagittal sinus and bilateral lateral and sigmoid sinuses.

A non-contrast CT of the head was done prior to lumbar puncture (LP) and was unremarkable. The opening pressure of CSF was 40 cm of water, and 30 ml was drained. The CSF examination was within normal limits. (Table 1) After the LP, the headache resolved, and the vision in the right eye improved, but there was no improvement in the left eye vision. Magnetic resonance imaging (MRI) and magnetic resonance venography (MRV) was done and showed extensive cerebral venous sinus thrombosis, including the superior sagittal sinus and bilateral lateral and sigmoid sinuses. (Fig. 1).

A bone marrow biopsy was performed considering the possibility of a myeloproliferative neoplasm, which revealed active erythropoiesis and myelopoiesis with prominent megakaryocytic hyperplasia. The biopsy was consistent with essential thrombocythemia. JAK2 V617F mutation was positive with a negative BCR-ABL. A multidisciplinary meeting was held between radiology, neurology, and hematology, and a formal diagnosis of CVST secondary to ET was made. The patient was initiated on aspirin 100 mg daily, hydroxyurea 500 mg twice daily, and rivaroxaban 15 mg twice daily for 21 days, followed by 20 mg daily. On a three-month follow-up, the patient's platelets normalized, his headache resolved completely; however, the blurring of vision persisted. The patient did not develop any anticoagulation related complications. Patient was advised for life-long anticoagulation in view of persistent risk factor (ET) for his CVST. However, after the first follow-up, the patient was lost to further follow-ups.

## 3 Discussion

Essential thrombocythemia is a myeloproliferative disorder with autonomous/primary thrombocytosis. In descending frequency, neurological complications associated with ET include cephalalgia, chronic paresthesia, dizziness or hypotension, visual disturbances, and tinnitus [14]. Venous thromboembolism associated with MPD presents commonly as DVT and uncommonly as splanchnic vein thrombosis [15]. CVST is relatively rare in association with MPD.

A literature review on PubMed using Boolean operator strategy of:

(Essential thrombocythemia) AND ((CVST) OR (Cerebral Venous sinus thrombosis)) performed on the 25th of November 2021 revealed 16 articles. Nine studies were excluded after screening the titles. Abstracts were reviewed for seven articles, and one more article was removed as it was not relevant [3,[9], [10], [11], [12], [13]]. Clinical characteristics, management, and outcomes of the reported cases of CVST secondary to ET are summarized in Table 2 which also summarizes our case in the last row.

**Table 2:** A literature review for the association of cerebral venous sinus thrombosis with essential thrombocytosis (NA: not available, Y: year, Mo: month, M: male, F: female).

Author (year)	Age (Y)/Gender (F/M)	Cell line involved	Highest platelets (*10^9^/L)	Site of thrombosis	CVST as initial presentation	Anticoagulation	Hydroxyurea	Aspirin	Other treatment	Prognosis
Jensen AW et al. (2007)	15, M	Platelets	1197	Left transverse sinus, sigmoid sinus, and vein of Labbe	Yes	Warfarin	Yes	Yes	NA	Follow-up after six months showed progressive recanalization of the left transverse sinus and his symptoms resolved.
Chen WB et al. (2018) Patient 1, 1^st^attack	33, M	Platelets	457–593	Superior sagittal sinus, straight sinus, and bilateral transverse sinus	Yes	LMWH followed by warfarin	Yes	Yes	NA	Good with cytoreduction and antithrombotic therapy until the patient discontinued medication after 4 years. Two months later, he experienced second attack.
Chen WB et al. (2018) Patient 1, 2nd attack	–	Platelets	181	Superior sagittal sinus, straight sinus, and bilateral transverse sinus. Multiple lacunar infarctions.	Yes	No	No	Yes	NA	NA
Chen WB et al. (2018) Patient 2	34, F	Platelets	350–593	Great cerebral vein, straight sinus right transverse sinus, and sigmoid sinus	Yes	LMWH Warfarin	No	Yes	Interferon-alpha	Good with cytoreduction and antithrombotic therapy in next six years
Chen WB et al. (2018) Patient 3	71, M	Platelets	460–759	Superior sagittal sinus, straight sinus, and bilateral transverse sinus	Yes	LMWH Warfarin	Denied Cytoreduction	Yes	NA	He denied cytoreduction. His symptoms improved slowly, and no thrombotic event was observed in the subsequent six years.
Chen WB et al. (2018) Patient 4, 1^st^attack	43, M	Platelets	456	Superior sagittal, right transverse, and sigmoid sinus	Yes	LMWH Warfarin	NA	NA	Selective thrombolysis and endovascular thrombectomy	Favorable long-term outcomes with antithrombotic and cytoreduction
Chen WB et al. (2018) Patient 4, 2nd attack	–	Platelets	352–506	–	Yes	LMWH Warfarin	Yes	Yes	Endovascular thrombectomy	–
Kurosawa H et al. (2009)	6, F	Platelets	680	Superior sagittal, right transverse sinus and sigmoid sinus	Yes	NA	Initially, HU was not administered. But later started due to recurrent thrombosis	Yes	NA	At 2 years follow up, long term HU therapy planned to prevent thrombosis until JAK 2 inhibitors are available.
Messouak O et al. (2007)	20, F	Leukocytes Erythrocytes Platelets	998	Superior longitudinal Sinus and Lateral Sinus	No	LMWH Warfarin	NA	NA	Antiedematous (acetazolamide). Allopurinol and hydroxycarbamide as a cytoreductive therapy	NA
Arai M et al. (2004)	52, F	Platelets	737	Superior sagittal sinus thrombosis, infarction of right frontal lobe	Yes	LMWH Warfarin	NA	Yes	NA	NA
Benmalek R et al. (2021)	39, M	Erythrocytes, Leucocytes with raised neutrophil count, Platelets	409	Left Circumflex Artery and Superior Sagittal Sinus	No	Intravenous Unfractionated Heparin Oral Vitamin K Antagonists	Yes	Yes	Ramipril Bisoprolol Furosemide Atorvastatin	Favorable progress after three weeks of treatment and progressive correction of hematological parameter
Our case	37, M	Platelets	1327	Superior Sagittal Sinus, Bilateral lateral and Sigmoid Sinus	Yes	Rivaroxaban	Yes	Yes	–	At 3 months follow up, there was no complaint of headache with normal platelet count. However, blurring of vision persisted.

The median age of presentation of previously published cases is 34 years, with a female preponderance (55%). This female preponderance is likely due to hormonal states conducive to thrombosis like pregnancy, puerperium, oral contraceptive use, and hormone replacement therapy [2,16]. Headache is the most common presentation. However, clinical features of raised ICP such as nausea, vomiting, or blurred vision are also reported [7,16]. The neuroradiological imaging studies show the involvement of multiple sinuses in ET-associated CVST. Superior sagittal sinus and transverse sinus are the most commonly involved sites of thrombosis. The review of articles reporting patients with CVST as an initial presentation of ET shows that the risk of thrombosis and platelet count are not congruent. However, all patients showed symptomatic benefit from anticoagulation and cytoreductive therapy, albeit the resolution of symptoms varied. The decreased platelets on follow-up in these patients may reflect the effects of increased consumption, anticoagulation, or cytoreduction [7].

In the acute presentation of CVST, parenteral anticoagulation with LMWH is recommended even in the presence of hemorrhage [2,7]. However, an oral anticoagulant like warfarin is recommended after initial anticoagulation with LMWH. The duration of anticoagulation depends upon the etiology of CVST [2]. New Oral Anticoagulants (NOACs) such as Rivaroxaban are being studied for their safety and efficacy in CVST. Currently, the data is limited to case reports, retrospective studies, and small trials. However, the results concerning efficacy seem to be similar to warfarin in CVST [[17], [18], [19]]. A recent systematic review has reported adequate efficacy and safety of NOACs compared to warfarin in CVST, with uncertainties persisting in dosing and duration of treatment [20]. Bose et al. reported the mean duration of 8.1 months for NOACs compared to 9.8 months with standard therapy [20]. However, current guidelines await more extensive randomized trials and meta-analyses on NOACs in CVST before adding them into its management guidelines [21]. Until more evidence is available, NOACs should be used in CVST in cases where warfarin use is not feasible, such as in our case where the patient could not monitor INR due to remote access. Some data support the benefits of selective thrombolysis and endovascular thrombectomy in patients with severe CVST and worsening neurological symptoms despite heparin use [3,22]. On imaging, patients developing cerebral venous congestion or cerebral edema also benefit from selective thrombolysis and endovascular thrombectomy [3,7,26]. The prognosis of CVST is better in women with identifiable risk factors than women with no identifiable risk factor or men [1].

The management of ET requires risk stratification. The role of low-dose aspirin as a prophylactic antithrombotic agent is established, though with an increased risk of minor bleeding [23,24]. Aspirin monotherapy is used in low and intermediate-risk diseases without cardiovascular risk factors. Patients with CVST associated ET are considered to have high-risk disease irrespective of their age [3,12]. Hydroxyurea is the drug of choice for cytoreduction. It is used with aspirin in high-risk diseases with a history of thrombosis or positive JAK2 mutation and intermediate-risk disease with cardiovascular risk factors. HU decreases the risk of early thrombotic events and decreases the leukocyte activation responsible for inducing a hypercoagulable state in ET [25,26]. There is a 3.5% increased risk of leukemia in ET patients treated with HU alone [27]. Due to its leukemogenic and mutagenic potential, hydroxyurea is administered only if the benefits outweigh the risk of acute leukemia [28]. The evidence on the role of direct oral anticoagulation (DOACs) in MPD is still not well-established [29,30].

The mainstay of treatment of CVST associated ET is anticoagulation, initially with intravenous heparin or oral warfarin, then later on with long-term low-dose aspirin, and cytoreduction with hydroxyurea (HU) [3,10,11,12]. The overall prognosis remains good with anticoagulation and cytoreduction. However, neurologic complications can persist [3]. Due to less mutagenic and more negligible pro leukemic effect, interferon-alpha is a substitute for HU in young, pregnant, and ET refractory to HU therapy.

## 4 Conclusion

In patients presenting with CVST and persistent thrombocytosis, myeloproliferative disorders should be investigated as a possible cause of thrombosis.

## Conflict of interest

None of the authors have any conflict of interest to declare.

## Financial disclosure

None.

## Consent

Written informed consent was taken from the patient before the submission of the article.

## Author contributions

QY: Case identification, literature review, manuscript writing.

HK and AK: Data collection, literature review, manuscript writing.

FA and ZY: Supervision, literature review, revisions and editing in the manuscript.

All authors reviewed and approved the final version of the manuscript.
